# Discordant prognosis of mismatch repair deficiency in colorectal and endometrial cancer reflects variation in antitumour immune response and immune escape

**DOI:** 10.1002/path.5894

**Published:** 2022-04-01

**Authors:** Mark A Glaire, Neil AJ Ryan, Marieke E Ijsselsteijn, Katarzyna Kedzierska, Sofia Obolenski, Reem Ali, Emma J Crosbie, Tjalling Bosse, Noel FCC de Miranda, David N Church

**Affiliations:** ^1^ Cancer Genomics and Immunology Group, Wellcome Centre for Human Genetics University of Oxford Oxford UK; ^2^ Division of Cancer Sciences, Faculty of Biology, Medicine and Health University of Manchester, St Mary's Hospital Manchester UK; ^3^ Division of Evolution and Genomic Medicine, Faculty of Biology, Medicine and Health University of Manchester, St. Mary's Hospital Manchester UK; ^4^ The Academic Women's Health Unit, Translational Health Sciences Bristol Medical School, University of Bristol Bristol UK; ^5^ Department of Pathology Leiden University Medical Center Leiden The Netherlands; ^6^ Department of Obstetrics and Gynaecology St Mary's Hospital, Manchester University NHS Foundation Trust, Manchester Academic Health Science Centre Manchester UK; ^7^ Oxford Cancer Centre, Churchill Hospital, Oxford University Hospitals Foundation NHS Trust Oxford UK; ^8^ Oxford NIHR Comprehensive Biomedical Research Centre, Oxford University Hospitals NHS Foundation Trust Oxford UK

**Keywords:** colorectal cancer, endometrial cancer, mismatch repair deficiency, microsatellite instability, immune response, immune escape

## Abstract

Defective DNA mismatch repair (dMMR) causes elevated tumour mutational burden (TMB) and microsatellite instability (MSI) in multiple cancer types. dMMR/MSI colorectal cancers (CRCs) have enhanced T‐cell infiltrate and favourable outcome; however, this association has not been reliably detected in other tumour types, including endometrial cancer (EC). We sought to confirm this and explore the underpinning mechanisms. We first meta‐analysed CRC and EC trials that have examined the prognostic value of dMMR/MSI and confirmed that dMMR/MSI predicts better prognosis in CRC, but not EC, with statistically significant variation between cancers (hazard ratio [HR] = 0.63, 95% confidence interval [CI] = 0.54–0.73 versus HR = 1.15, 95% CI = 0.72–1.58; *P*_INT_ = 0.02). Next, we studied intratumoural immune infiltrate in CRCs and ECs of defined MMR status and found that while dMMR was associated with increased density of tumour‐infiltrating CD3^+^ and CD8^+^ T‐cells in both cancer types, the increases were substantially greater in CRC and significant only in this group (*P*_INT_ = 4.3e‐04 and 7.3e‐03, respectively). Analysis of CRC and EC from the independent Cancer Genome Atlas (TCGA) series revealed similar variation and significant interactions in proportions of tumour‐infiltrating lymphocytes, CD8^+^, CD4^+^, NK cells and immune checkpoint expression, confirming a more vigorous immune response to dMMR/MSI in CRC than EC. Agnostic analysis identified the IFNγ pathway activity as strongly upregulated by dMMR/MSI in CRC, but downregulated in EC by frequent *JAK1* mutations, the impact of which on IFNγ response was confirmed by functional analyses. Collectively, our results confirm the discordant prognosis of dMMR/MSI in CRC and EC and suggest that this relates to differences in intratumoural immune infiltrate and tumour genome. Our study underscores the need for tissue‐specific analysis of cancer biomarkers and may help inform immunotherapy use. © 2022 The Authors. *The Journal of Pathology* published by John Wiley & Sons Ltd on behalf of The Pathological Society of Great Britain and Ireland.

## Introduction

The DNA mismatch repair (MMR) system plays a critical role in suppression of mutagenesis and cancer [1]. Defective DNA mismatch repair (dMMR) occurs with variable prevalence across cancer types, although most commonly in colorectal and endometrial cancer (CRC and EC), in which it is found in 10–15% and 20–25% of cases respectively [2, 3]. Mechanistically, dMMR arises either as a consequence of inherited defects in MMR genes—a condition known as Lynch syndrome (LS)—or as a somatic event from *MLH1* promoter methylation or biallelic somatic MMR gene mutations. Irrespective of its cause, it leads to a failure to correct base mispairs and small insertion–deletion (indel) mutations incorporated during DNA replication, leading to an elevated tumour mutational burden (TMB), and slippage at repetitive DNA microsatellites, a phenomenon referred to as microsatellite instability (MSI) [2, 3]. Early studies suggested that early‐stage colorectal cancers (CRCs) with dMMR had a favourable outcome, a finding which has been confirmed in other series [4]. The subsequent demonstration that dMMR CRCs commonly display a dense lymphocytic infiltrate [5] provided a plausible explanation for their improved prognosis, and has led to a broadly accepted model in which dMMR causes elevated TMB, and an increase in the number of mutated neoantigens recognised as nonself by cytotoxic T‐cells, inducing a tumour suppressive cytolytic immune response [6]. This model is supported by recent data showing that LS‐associated dMMR tumours have higher TMB and denser T‐cell infiltrate than sporadic dMMR tumours of the same type [7, 8], and that (at least in the case of CRC), this is reflected in a seemingly better outcome for LS dMMR cases [7]. Of course, the fact that all such cancers have grown to the extent that they are diagnosed demonstrates that some degree of immune escape has occurred. The mechanisms by which this occurs are multiple and an active area of research, but it is clear that mutations in antigen presentation components and immunomodulatory signalling pathways play a pivotal role, as does upregulation of immunosuppressive immune checkpoint molecules such as CTLA4, PD1, and PDL1 [6, 9, 10]. Targeting of these immune checkpoints in otherwise treatment‐refractory dMMR metastatic cancers results in frequent and, in some cases, sustained responses [11], leading the US Food and Drug Administration (US FDA) to license the use of anti‐PD1 therapy for this molecular subset irrespective of histology; the first tumour type agnostic biomarker approved for such an indication [12].

While the molecular and clinical impact of dMMR has been intensively investigated, current understanding of its variation across cancers remains far from complete. Perhaps the most obvious example is between the cancer types in which dMMR is most common—colorectal and endometrial [2, 3]. In both cases, dMMR/MSI tumours display hypermutation (often defined as TMB greater than 10 mutations/Mb), and increased density of cytotoxic T lymphocytes when compared to their proficient MMR (pMMR) / microsatellite stable (MSS) counterparts [13, 14, 15, 16]. However, while formal comparison has not been performed, most studies of the prognosis of dMMR EC have not reported the favourable prognosis seen in CRC [17], and some have even suggested a worse outcome than pMMR/MSS ECs, with a similarly low burden of copy number alterations [18]. This discordance is intriguing, as ultramutated (typically >100 mutations/Mb) tumours with *POLE* exonuclease domain mutations display excellent prognosis in both tumour types [19, 20]. It is also not merely of academic interest, because understanding the underlying mechanisms could reveal novel therapeutic targets in dMMR EC to improve the outcome in this challenging subset. In this study we sought to define the variation in prognosis of dMMR between CRC and EC by meta‐analysis, and to delineate the underpinning immunological and molecular mechanisms. Our results provide new insights into the tissue‐specific variation of dMMR and the immune response to hypermutation.

## Materials and methods

### Ethical approval

The Biomarkers Of Lynch syndrome Tumours (BOLT) study was sponsored by the University of Manchester and approved by the North West Greater Manchester Research Ethics Committee (ref: 16/NW/0164).

Ethical approval for analysis of human tumour samples used in this study was given by committees at all participating institutions; approval for integrated analysis of datasets was provided by REC reference 18/SC/0533.

### Design and study cohorts

Studies for meta‐analysis were identified by PubMed search using the terms ‘colorectal’ OR ‘endometrial’ AND ‘mismatch repair’ OR ‘microsatellite instability’ AND ‘prognosis,’ limiting results to clinical trials published between 2000 and 2021. Cases with defined mismatch repair status (pMMR, dMMR sporadic, dMMR Lynch) for immunoprofiling were assembled from the BOLT study [21] and retrospective population‐based cohorts curated by the University of Manchester (UK) and the Leiden University Medical Center (NL).

### Determination of dMMR/MSI and Lynch syndrome status in study cohorts

MMR status in the retrospective CRC and EC cohorts was determined by immunohistochemistry as reported previously [22]. Following microwave antigen retrieval (in 10 mm Tris‐EDTA buffer, pH 9.0), formalin‐fixed paraffin‐embedded (FFPE) sections were incubated overnight with primary antibodies against MLH1 (clone ES05, 1:100; Dako), MSH2 (clone FE11, 1:200, Dako, Santa Clara, CA, USA), MSH6 (clone EPR3945, 1:800, Genetex, Irvine, CA, USA), all at room temperature, or PMS2 (clone EP51, 1:75, Dako) at 4 °C. Sections were then incubated for 15 min at room temperature with Envision FLEX+ Linker (Dako), followed by 30 min with secondary antibody (Poly‐HRP‐GAM/R/R; DPV0110HRP; ImmunoLogic, Duiven, The Netherlands), and colour developed using DAB before counterstaining and mounting. MMR loss was defined as complete loss of epithelial staining for ≥1 protein in the presence of positive stromal or immune cells. Tumour MSI status was determined by Promega MSI analysis system (v. 1.2, Promega, Madison, WI, USA) as reported previously [22]. Tumours with instability in ≥2 of 5 mononucleotide repeat markers were defined as being microsatellite instability‐high (MSI), whereas those showing no instability or instability at a single repeat were classified as MSS. Confirmation of Lynch syndrome was made by Regional Genetics Laboratories following testing of constitutional DNA for pathogenic MMR gene variants (InSiGHT Class IV or V) [23].

### Multispectral immunofluorescence staining

Tumour infiltrating immune cells were quantified by multispectral immunofluorescence (IF) as described previously [24]. FFPE slides were deparaffinised before antigen retrieval using citrate buffer. The slides were then incubated overnight with primary antibodies for FoxP3 (236A/E7, 1:25, ThermoFisher Scientific, Waltham, MA, USA) and CD8 (4B11, 1:50, ThermoFisher Scientific). Following washes with phosphate‐buffered saline (PBS) supplemented with 0.05% Tween, slides were incubated for a further 1 h with the appropriate secondary fluorophore antibodies (CFF633 goat antimouse IgG1, 1:100, [Avantor, Radnor, PA, USA] and CF555 goat anti IgG2b, 1:100, [Sigma‐Aldrich, St. Louis, MO, USA] for FoxP3 and CD8 detection, respectively). The slides were then washed again, then incubated with conjugated antibodies (primary antibody with fluorophore) for CD3 (D7A6E, 1:50, Cell Signaling Technology, Beverly, MA, USA) and pan‐cytokeratin (Pankeratin (C11), 1:50, Cell Signaling Technology and Cytokeratin, AE1/AE3, 1:50, ThermoFisher Scientific) for 6 h. The slides were then washed again, DAPI applied as a nuclear stain and the slides mounted using ProLong Gold (ThermoFisher).

### Image acquisition and analysis

Multispectral IF‐stained slides were imaged using the Vectra Polaris imaging system (Perkin Elmer, Waltham, MA, USA). Following initial whole slide scans, high‐power (40×x objective magnification) images were taken for analysis from representative sections (two from the tumour centre and one from the invasive margin). Tissue was segmented into epithelia and stroma by training on DAPI and cytokeratin stains and the following cell populations defined and quantified: total T‐cells (CD3^+^), cytotoxic T‐cells (CD3^+^CD8^+^), and T regulatory cells (CD3^+^FoxP3^+^).

### TCGA analysis

MC3 MAFs containing curated TCGA colorectal (COADREAD) and endometrial (UCEC) cancer whole exome sequencing (WES) data were downloaded from the NIH Genomic Data Commons (https://gdc.cancer.gov/about-data/publications/mc3-2017) along with tumour MSI status determined by testing of microsatellite markers [13, 14]. Germline MMR gene mutations were identified by analysis of controlled‐access BAMs for pathogenic variants [23]. Controlled FASTq files for RNAseq data were downloaded from the NIH GDC data portal (https://portal.gdc.cancer.gov/) and processed by Salmon (see Supplementary materials and methods) to obtain relative transcript abundance (Transcripts Per Million—TPM) for downstream analysis. Estimates of tumour‐infiltrating immune cell populations and related data were downloaded from the supplementary material of Thorsson *et al* [25]. To calculate absolute estimates of immune cell populations, proportional estimates were multiplied by leucocyte fraction, the latter being an estimate of the total fraction of the bulk tumour due to immune cells. Methylation data for TCGA cases were downloaded from the National Institutes of Health (NIH) Genomic Data Commons (GDC). Probes mapping to *MLH1*, which were most differentially methylated according to tumour MSI status were identified using the TCGAbiolinks package (see Supplementary materials and methods).

### Cell lines

Human endometrial adenocarcinoma cell lines were a kind gift from Konstantin Dedes (University of Zurich, Switzerland) and Britta Weigelt (previously Cancer Research UK, Lincoln's Inn Fields UK) or purchased from the European Collection of Authenticated Cell Cultures or the JCRB cell bank. The MSI status was determined using the Promega MSI analysis system and *JAK1* mutation by whole exome sequencing (WES) done in‐house or by the Cancer Cell Line Encyclopedia: https://depmap.org/portal/ccle/. Cell lines were cultured under standard conditions. Full details are provided in the Supplementary materials and methods.

### Interferon‐gamma stimulation

Cells for interferon‐gamma (IFN‐γ) stimulation were plated at 1×10^6^ cells in 25 cm^2^ cell culture flasks, grown to 70–80% confluency, and serum starved for 24 h before treatment with IFN‐γ (554617, BD Biosciences, Franklin Lanes, NJ, USA) at 75 ng/ml or an equivalent volume of PBS for 16 h before cell lysis and protein collection.

### Western blotting

Protein lysis and western blotting were performed according to standard methods. Full details, including antibody clones and concentrations, are provided in the Supplementary materials and methods.

### Statistical analyses

Continuous variables were analysed using the nonparametric Mann–Whitney U test, given the lack of normal data distribution, with interaction testing performed using aligned‐rank analysis of variance (ANOVA). Categorical variables were analysed using Fisher's exact test. Meta‐analysis of trial data was performed by both fixed and random effects models using inverse variance weighting. Differentially expressed gene analyses and dysregulated processes/pathways, were identified by DESeq2 and clusterProfiler, respectively (see Supplementary materials and methods). The association of candidate immune escape mutations with tumour IFNγ pathway activity was analysed by multiple linear regression including tumour type and MSI status as covariables. All statistical analyses were performed in R, v. 4.0.1 (https://cran.r-project.org), using the packages ‘meta,’ ‘ggplot2,’ ‘DESeq2,’ ‘clusterProfiler,’ ‘TCGAbiolinks,’ and ‘MAFtools’ (see Supplementary materials and methods for sources). Statistical tests were two‐sided, and hypothesis testing was performed at the 5% significance level.

## Results

### Prognostic value of dMMR/MSI varies between CRC and EC

**Figure 1:**
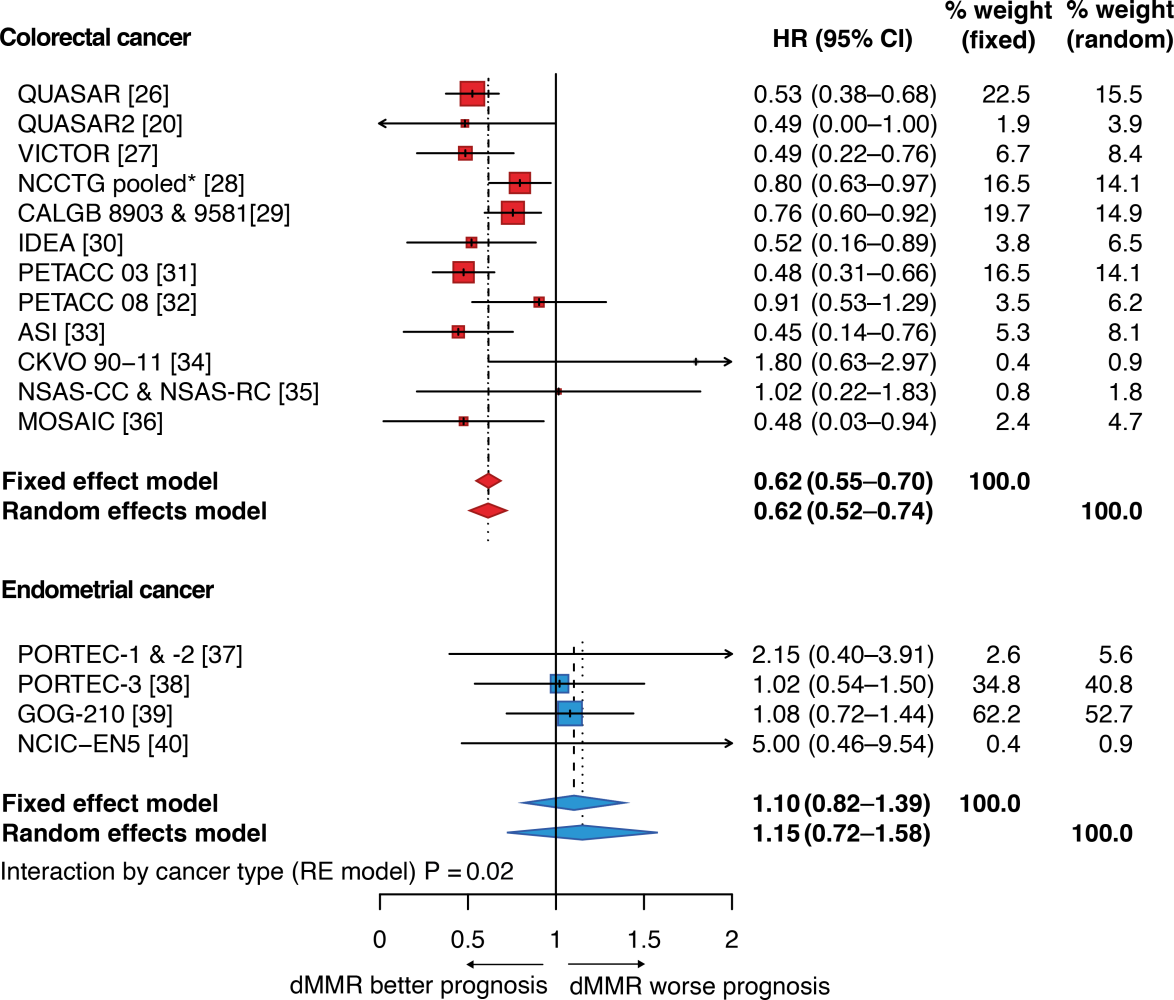
Association between MMR status and disease‐free survival (DFS) in colorectal and endometrial cancer. Forest plot showing meta‐analysis of clinical trials that have examined association of dMMR with DFS in CRC and EC (details in Materials and methods). Corresponding plot for overall survival (OS) is provided as supplementary material, Figure S1. *Pooled analysis includes Mayo Clinic and North Central Cancer Treatment Group (NCCTG) trials 78‐48‐52, 84‐46,52/Intergroup 0035, 89‐46‐51, 79‐46‐04, 87‐46‐51, 91‐46‐53, Federation Francophone de la Cancerologie Digestive (FFCD) 8802, Gruppo Italiano Valutazione Interventi in Oncologia (GIVIO), National Cancer Institute of Canada (NCIC) C03, NSABP C‐01, C‐02, C‐03, and C‐04.

The robust association of dMMR with reduced recurrence risk in stage II CRC is reflected in its inclusion as a prognostic biomarker in clinical guidelines. However, similar analysis of EC suggests that dMMR carries either no prognostic import, or predicts worse outcome [17]. To confirm and quantify this, we did a meta‐analysis of published studies that have addressed this question. Limiting our inclusion to early‐stage (i.e. nonmetastatic) disease and to clinical trials (which provide a higher level of evidence than nonexperimental cohorts), we identified 12 CRC [20, 26, 27, 28, 29, 30, 31, 32, 33, 34, 35, 36] and 4 EC [37, 38, 39, 40] publications that met our search criteria (see Materials and methods and the PRISMA diagram shown in supplementary material, Figure S1 for details) reporting the analysis of 27 and 5 trials, respectively. Details of these studies are summarised in supplementary material, Table S1. Multivariable‐adjusted hazard ratios (HRs) for disease‐free survival (DFS) and overall survival (OS) were extracted from the study publications and pooled by fixed‐effects (FE) and random‐effects (RE) meta‐analysis. As expected, dMMR was associated with a substantial and highly significant improvement in DFS in CRC (HR_RE_ = 0.62; 95% CI = 0.55–0.70%; *p* < 1e‐04), with moderate heterogeneity across studies (I2 = 47%, τ2 = 0.015, *p* = 0.036) (Figure 1). In contrast, similar analysis of EC revealed no significant association of dMMR with tumour recurrence (HR_RE_ = 1.15; 95% CI = 0.72–1.58; *p* = 0.23), without significant between‐study heterogeneity (I2 = 31%, τ2 = 0.0571, *p* = 0.23) (Figure 1). Formal testing confirmed that this variation by tumour type was statistically significant (*p* = 0.02). Similar results were obtained for meta‐analysis of OS (supplementary material, Figure S2). Thus, dMMR is associated with better prognosis in early‐stage CRC, but not in EC.

### dMMR is associated with greater increase in T‐cell infiltrate in CRC than in EC

**Figure 2:**
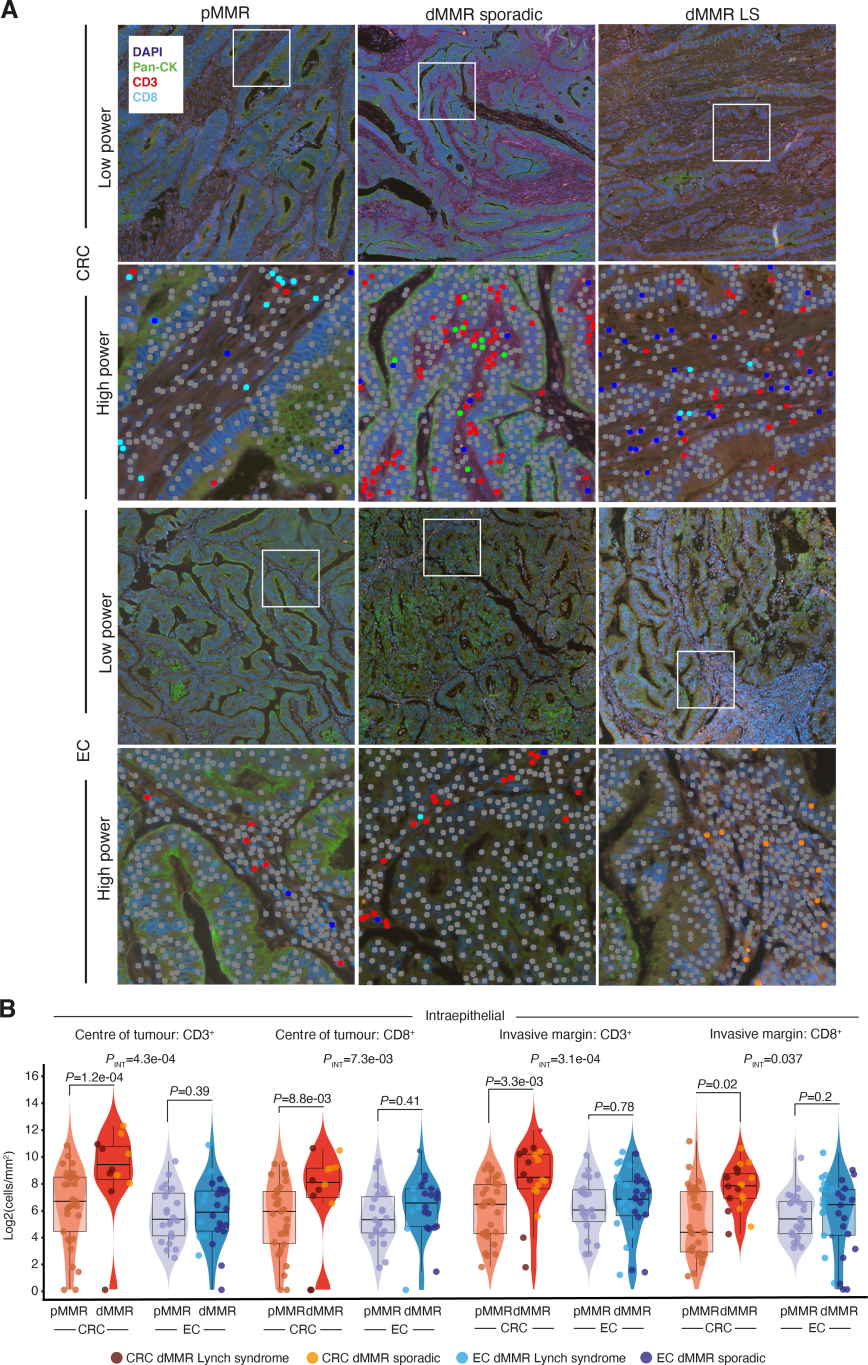
Intratumoural CD3^+^ and CD8^+^ cell infiltrate by MMR status and cancer type. (A) Representative multiplex co‐immunofluorescence images from mismatch repair‐proficient (pMMR) and mismatch repair‐deficient (dMMR) tumours of sporadic (*MLH1* methylated) and Lynch Syndrome (LS) aetiology. High‐power magnification images illustrate automated quantification of tumour‐infiltrating immune cells. (B) Quantification of density of intraepithelial CD3+ and CD8+ cells in the tumour centre and invasive margin. Corresponding quantification for intrastromal lymphocytes is shown in supplementary material, Figure S2. Boxplots indicate sample median (thick line) interquartile ranges. Comparison between groups used a Mann–Whitney *U* test; tests for interaction used aligned rank ANOVA.

Work from our group and others' has shown that dMMR is associated with increased intratumoural cell infiltrate in both CRC and EC [15, 16, 20]; however, whether this differs in magnitude and type between these cancer types is unknown. The discordant prognostic value of dMMR in CRC versus EC led us to speculate on this possibility. To test this, we assembled a large cohort of CRCs and ECs of defined MMR status, including both hereditary (LS) and sporadic (*MLH1* methylation) causes of dMMR, and quantified the density and localisation of lymphocytic infiltrate by multispectral co‐immunofluorescence (see Materials and methods). Given the known association of *POLE* exonuclease domain mutations with increased tumour T‐cell infiltrate [15, 20], we excluded these from our analysis. When compared with MMRp tumours, median intraepithelial (IE) CD3^+^ and cytotoxic CD8^+^ T‐cell densities were numerically greater in dMMR tumours in both CRC and EC, with LS tumours tending to have denser infiltrate, in keeping with previous reports [7, 8, 41] (Figure 2A,B). However, while in CRC these increases were substantial and highly statistically significant, in EC any differences were far more modest and not statistically significant, reflected in statistically significant MMR*tumour type interactions for both markers (*P*_INT_ = 4.3e‐04 and 7.3e‐03, respectively) (Figure 2B, supplementary material, Table S2). Analysis of the tumour invasive margin revealed similar findings (*P*_INT_ = 3.1e‐04 and 0.037, respectively) (Figure 2B). Differences in intrastromal CD3+ and CD8+ infiltrate showed similar trends but were more modest (supplementary material, Figure S3A). No significant difference between groups was detected for either intraepithelial and intrastromal FoxP3+ cell infiltrate (supplementary material, Figure S3B).

### TCGA analysis confirms variable immune response against MSI between CRC and EC and identifies IFNγ response as a key discordant pathway

**Figure 3:**
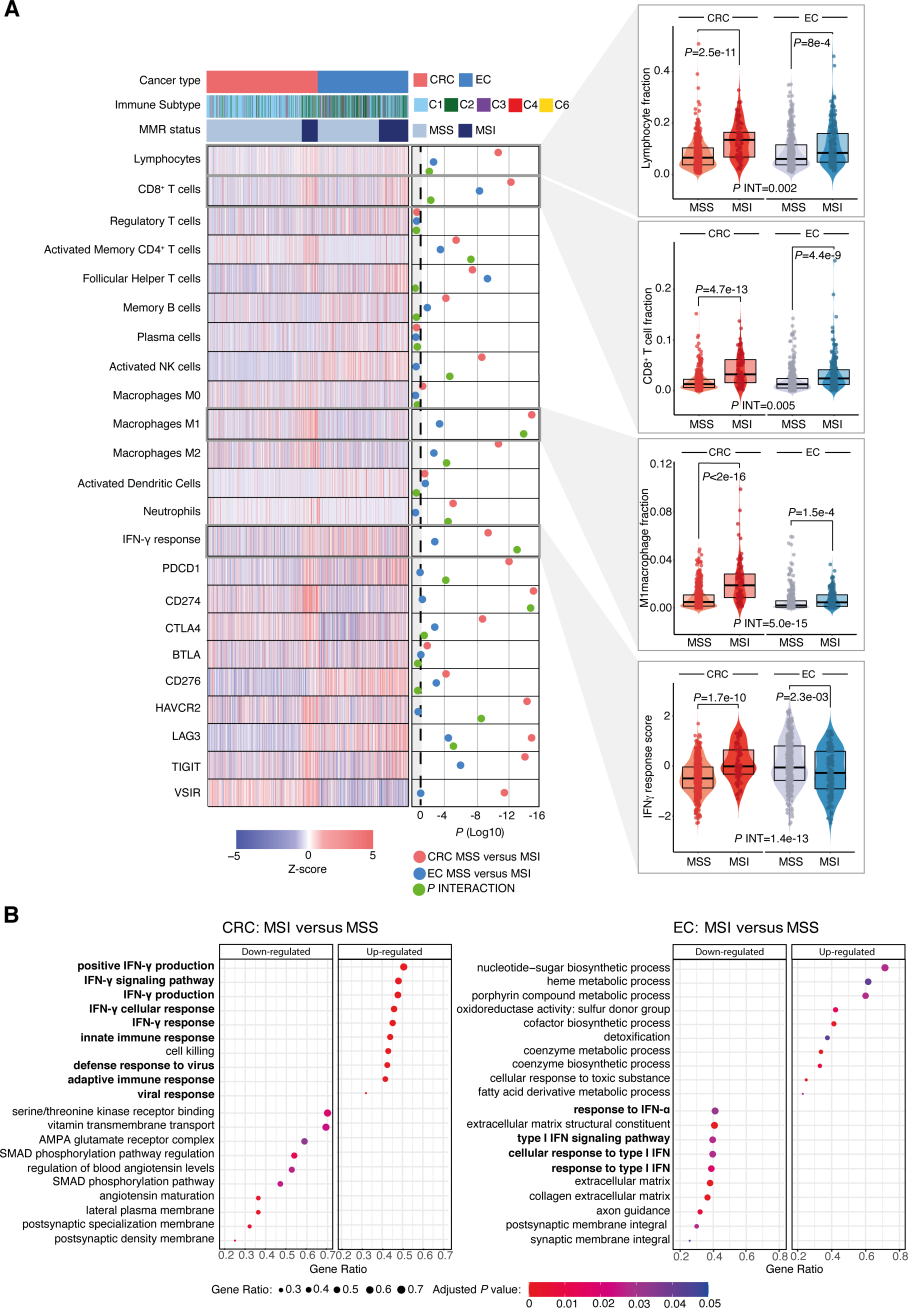
Intratumoural immune cell infiltrate, immune checkpoint expression, and most highly dysregulated pathways by MMR status and cancer type. (A) Heatmap showing relative tumour proportion of tumour‐infiltrating immune cells, IFNγ response score, and expression of immune checkpoints by cancer type, MMR status, and immune subgroup (C1–C6) according to the classification in Thorsson *et al* [25]. Corresponding scatterplots showing immune checkpoint expression are shown in the supplementary material, Figure S3. (B) Ten most upregulated and downregulated gene sets in MSI versus MSS tumours identified by gene set enrichment analysis. Immune‐related gene sets are highlighted in bold. Comparison between MSI and MSS tumours within cancer types in (A) used a Mann–Whitney *U* test; tests for interaction used aligned rank ANOVA. CRC, colorectal cancer; EC, endometrial cancer; MSI, microsatellite unstable; MSS, microsatellite stable.

We sought to validate and extend these results by analysis of tumours from TCGA [13, 14], taking MSI as a broadly accepted surrogate for MMR deficiency. Using CIBERSORT estimates of tumour immune cell fractions from the Thorsson TCGA pan‐cancer immune study [25] (in which data underwent rigorous QC and extensive batch correction), we first confirmed that MSI was associated with a substantially greater increase in intratumoural lymphocyte fraction, and CD8+ cell fraction in CRC than EC, with statistically significant interaction in both cases (*P*_INT_ = 0.002 and *P*_INT_ = 0.005, respectively) (Figure 3A). Similar variation and statistically significant interactions were also observed for CIBERSORT estimates of CD4^+^ cells, activated NK cells, M1 and M2 macrophages, and neutrophils (Figure 3A), and expression of immune checkpoints PDCD1 (PD1), CD274 (PDL1), CTLA4, HAVCR2, LAG3, TIGIT, and VSIR determined by RNAseq (Figure 3A, supplementary material, Figure S4). We next took a hypothesis‐free approach to examine the consequences of dMMR/MSI between tumour types through gene set enrichment analysis (GSEA), beginning with CRC. Strikingly, among the 10 most enriched gene sets in MSI CRCs, nine involved an immune or antiviral response, including five corresponding to the IFNγ response, a pivotal player in antitumour immunity [42], and a central determinant of response to immune checkpoint blockade [43] (Figure 3B). In contrast, similar analysis of EC not only failed to demonstrate enrichment of immune system processes among MSI tumours, but also revealed significant downregulation of multiple IFNα gene sets in this subgroup (Figure 3B). Further analysis using a published IFNγ gene signature [25] revealed striking variation by cancer type and MSI status. While IFNγ pathway activity was, in general, lower in CRC than in EC, MSI CRCs demonstrated substantially, and highly significantl greater pathway activity compared to MSS CRCs (*p* = 1.7e‐10). In contrast, among ECs the opposite was the case; that is, MSI tumours had significantly lower IFNγ pathway activity (*p* = 2.3e‐03). Formal testing confirmed this interaction was highly statistically significant (*P*_INT_ = 1.4e‐13) (Figure 3A).

### Recurrent *JAK1* frameshift mutations disrupt IFNγ signalling in MSI EC

**Figure 4:**
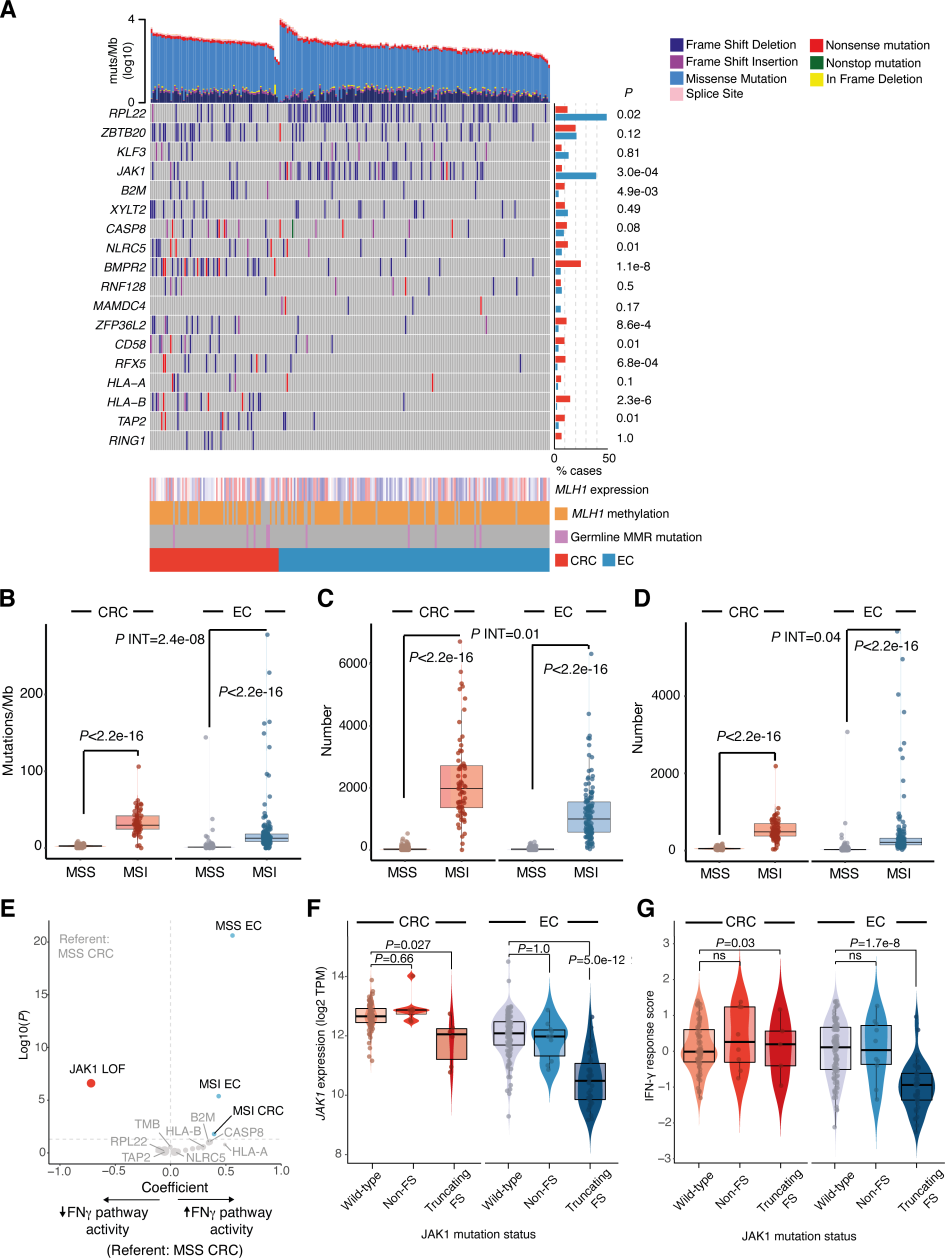
Tumour mutation burden, immune escape mutations, and interferon gamma pathway activity. (A) Oncoprint showing tumour mutation burden stratified by type, immune escape mutations (list from [44]), MLH1 expression, and promoter methylation and germline MMR gene mutation. Bars to the right indicate the proportion of cases with loss of function (LOF) mutations, defined as a truncating point or frameshift variants. (B–D) Tumour nonsilent mutation rate (B), predicted indel neoantigen burden (C), and single nucleotide variant (SNV) neoantigen burden (D) according to cancer type and MMR status. (E) Multiple linear regression for predictors of IFNγ response. Regression included tumour type and MMR status, and candidate immune escape mutations shown in (A). (F,G) Relationship between *JAK1* mutation and (F) JAK1 expression and (G) IFNγ response score in MSI CRC and EC. Comparison of LOF mutation frequency in (A) used Fisher's exact test. Comparison between groups in (C, D) used Mann–Whitney *U* tests; tests for interaction used aligned rank ANOVA. CRC, colorectal cancer; EC, endometrial cancer; FS, frameshift mutation; MSI, microsatellite unstable; MSS, microsatellite stable.

**Figure 5:**
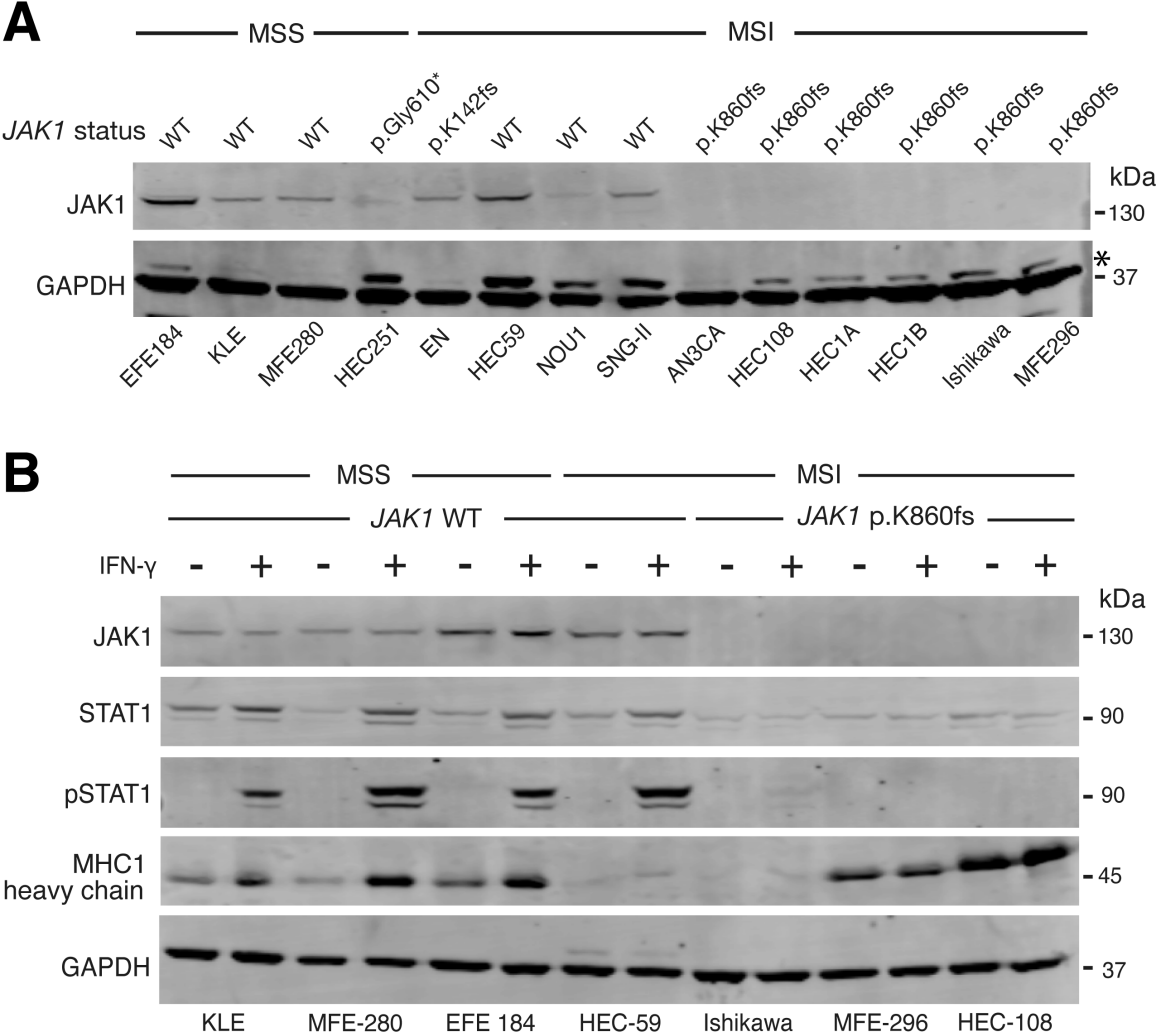
*JAK1* mutation, protein levels and IFNγ response in endometrial cancer cell lines. (A) Immunoblot showing MMR status, *JAK1* mutation, and JAK1 protein levels in a panel of 14 endometrial cancer cell lines. (B) Immunoblot showing downstream STAT1 induction, activating Y701 phosphorylation and HLA class I heavy chain levels in response to recombinant IFNγ (75 ng/ml for 16 h) in endometrial cancer cell lines according to *JAK1* truncating p. K860fs mutation and JAK1 protein loss. Molecular weights of standards are shown to the right of the panels. Further details including antibodies used, are provided in Materials and methods. MSS, microsatellite stable; MSI, microsatellite unstable. * In panel (A) indicates nonspecific bands at higher molecular weight than GAPDH loading control.

We used the TCGA series to investigate whether the variation in immune response between dMMR/MSI CRC and EC reflected underlying differences in tumour biology and immunogenicity. TMB and predicted single nucleotide variant (SNV) and indel neoantigen burden (predictors of intratumoural immune infiltrate) were elevated in MSI tumours of both types. However, the increases were significantly greater in CRC (*P*_INT_ = 2.48e‐08 to *P*_INT_ = 0.040) (Figure 4A–D). These differences could not obviously be explained by differences in *MLH1* promoter methylation or expression (Figure 4A, supplementary material, Figure S4), or secondary mutations in DNA repair genes (data not shown). Further analysis of a recently published set of genes implicated in immune escape [44] revealed notable variation between MSI tumours according to the tissue of origin. Genes enriched for loss of function (LOF) mutations in MSI CRC versus MSI EC included components of the MHC class I antigen presentation pathway, including *HLA‐B*, *B2M*, *NLRC5*, and *TAP2* (*p* = 0.01 to *p* = 2.3e‐06, Fisher's exact test) (Figure 4A). While only two immune escape genes—*JAK1* and *RPL22*—were more commonly subject to LOF mutations in MSI EC than MSI CRC, both were mutated at high frequency (23.8 and 31.9% cases, respectively, *p* = 3.0e‐04 and *p* = 0.02, respectively, Fisher's exact test) (Figure 4A), with the majority of *JAK1* mutations being a recurrent frameshift at codon 860 (p.K860Nfs*16). We used multiple regression to test whether these differences could account for the variation in IFNγ pathway activity between MSI CRC and EC. Interestingly, other than the expected associations with tumour type and MSI status, the only significant predictor of IFNγ response were *JAK1* LOF mutations, which predicted substantially reduced activity (β = −0.72, *p* = 2.48e‐07) (Figure 4E). Interestingly, reduced JAK1 expression and IFNγ were only detected in association with truncating frameshift mutations; predominantly the codon 860 variant (Figure 4F,G). JAK1 is a tyrosine kinase that binds IFN cell surface receptors to coordinate phosphorylation, oligomerization, and nuclear translocation of the transcription factor STAT1 [42], thus mediating type I and II IFN signalling. *JAK1* mutations have been reported in dMMR EC and highlighted as a likely mechanism of immune escape [45, 46]. However, these studies did not perform mechanistic analysis, and an early functional analysis of *JAK1* mutations in gynaecological cancers included only two EC cell lines and did not account for MMR status [47]. To study the impact of *JAK1* mutations in detail, we screened a panel of EC cell lines of known MSI and *JAK1* mutation status. *JAK1* LOF mutations were detected in eight lines, seven of which were MSI—the single other line being HEC‐251, which carries a pathogenic *POLE* mutation. Six of these LOF mutations were the recurrent p.K860Nfs*16 frameshift, with several mutant lines harbouring more than one truncating mutation, suggesting compound heterozygosity, or demonstrating mutant allele fractions consistent with loss of heterozygosity of the wildtype allele. Immunoblot analysis confirmed JAK1 protein expression in all four MSS cell lines (although expression was reduced in HEC251 cells) and all three MSI cell lines lacking JAK1 mutations (Figure 5A). In contrast, six of seven MSI cell lines with *JAK1* frameshift mutations showed loss of JAK1 protein, including all lines with the p.K860Nfs*16 variant (Figure 5A). Immunoblotting confirmed that JAK1 loss was associated with the absence of induction and phosphorylation of STAT1 and lack of upregulation of MHC class I heavy chain in response to IFNγ, consistent with functional loss of IFNγ pathway signalling (Figure 5B). Thus, the enrichment of JAK1 truncating frameshift mutations in dMMR/MSI EC at least partly explains their lack of IFNγ response, and may also partly explain the absence of a prognostic benefit of MMR loss in this tumour type.

## Discussion

In this study we demonstrated that the prognostic value of dMMR varies between the two tumour types in which it is most common—colorectal and endometrial—and that this discordance correlated with quantitative and qualitative differences in intratumoural immune cell infiltrate, and the underlying tumour genome. Furthermore, we identified the IFNγ response as a critical pathway upregulated in dMMR CRC, and downregulated in dMMR EC as a consequence of *JAK1* LOF mutations.

While the favourable prognosis of early‐stage dMMR CRC is well‐recognised, studies in EC have yielded conflicting results, reflected in a meta‐analysis that was inconclusive owing to marked interstudy heterogeneity [17]. By meta‐analysis of trial data, we show clear, statistically significant variation in the prognostic value of dMMR between these tumour types, with no detectable improvement in the latter. Motivated by this observation, we performed what is, to the best of our knowledge, the first comparison of the impact of dMMR/MSI on intratumoural infiltrate between CRC and EC. While as expected [7, 8, 15, 20], dMMR was associated with increased T‐cell infiltrate in both cancer types, the increase was markedly greater in CRC, evidenced by a statistically significant interaction. This discordance was mirrored by similar variation, (and statistically significant interactions), in tumour infiltration by multiple additional immune cells, and the IFNγ response—a key player in antitumour immunity [42] and response to immune checkpoint blockade [43]. Strikingly, while dMMR was associated with strong upregulation of the IFNγ pathway in CRC, in EC the opposite was the case. This appeared partly due to enrichment of *JAK1* truncating frameshift mutations, which were associated with reduced IFNγ response in human cancers, loss of IFN signalling in cell lines, and which have recently been shown to predict lack of response to immunotherapy in EC [48]. However, this did not appear to be the sole explanation, because dMMR ECs lacking *JAK1* mutations also demonstrated reduced IFNγ pathway activity compared to MMRp ECs. The possibility of hidden confounders means that cross‐cancer comparisons of RNAseq data must be interpreted cautiously, even following careful batch effect correction. Nevertheless, in general, IFNγ pathway activation appeared greater in EC than CRC, both across the whole cohorts, and among the majority of MMRp/MSS tumours. Speculatively, this difference may account for the apparent selection for *JAK1* LOF mutations in dMMR EC, and possibly even the partial sensitivity of MMRp ECs to immune checkpoint inhibition [49], in contrast to the refractoriness of MMRp CRC to such therapy. Other dMMR/MSI‐associated immune escape mutations that varied in frequency between cancer types included *RPL22*, which was enriched in EC, and the antigen processing and presentation pathway components *B2M*, *NLRC5*, *TAP2*, and *HLA‐B*, which were more commonly disrupted in CRC. While previous reports have noted variation in the type and frequency of somatic mutations between dMMR/MSI according to the site of origin, ours is the first to demonstrate similar variation in the genetic mechanisms of immune escape. It is also the first to demonstrate the discordance in TMB and predicted neoantigen burden between dMMR/MSI tumours of the colon and endometrium; an observation which did not appear to relate to differences in *MLH1* promoter methylation or expression. Understanding how these results relate to the differing immunological milieu between the immune‐rich human colon and the immune‐privileged endometrium, and the other factors that underpin these differences, will be important topics for future study, as will their utility as prognostic markers and predictors of immunotherapy benefit. Similar efforts also appear merited in other cancer types: while a recent study revealed better outcome of gastric cancers with dMMR/MSI (~8% cases) [50], the modest prevalence of dMMR/MSI in other cancers has precluded definitive conclusions on its prognostic value or immunological correlates.

Our study has limitations. The large number of tumours we analysed precluded the use of costly methods for detailed immunophenotyping. Consequently, it is unclear if other immune cell types (e.g. myeloid lineages and rarer T‐cell populations) and tumour PDL1 status display similar variation by MMR status and cancer type as the markers we assessed. Given that these markers have been shown to predict prognosis and/or benefit from immunotherapy, it will be important to address this in future studies. Furthermore, the formalin fixation of these retrospectively selected clinical samples prevented in‐depth genomic or transcriptomic interrogation. Similarly, TCGA samples permitted only analysis of the bulk transcriptome, meaning that subtle, epithelial‐specific perturbations could well have been missed. The use of MSI as a surrogate for dMMR in the TCGA cases is another limitation, as the correspondence between the two is less reliable in EC than in CRC, particularly in the minority of cases due to *MSH6* or *MSH2* gene defects. Finally, the correlative nature of our analyses means that, with the exception of *JAK1* mutation, the mechanistic underpinnings of our results await definition through functional studies.

To conclude, our study provides further evidence that the consequences of genetic alterations in cancer depend upon the tissue context in which they occur. Recognising this, and understanding the underpinning mechanisms, will be critical for delivery of precision medicine and immunooncology over the coming years.

## Supporting information


Supplementary materials and methods
**Figure S1.** PRISMA diagram showing search strategy used for identification of studies for inclusion in meta‐analysis
**Figure S2.** Deficient DNA mismatch repair predicts improved overall survival in colorectal but not endometrial cancer
**Figure S3.** Intrastromal CD3+ and CD8+, and intraepithelial and intrastromal FoxP3+ cell infiltrate by tumour type and MMR status
**Figure S4.** Immune checkpoint expression by cancer type and MSI status
**Figure S5.** MLH1 promoter methylation and expression by cancer type and MSI status
**Table S1.** Details of studies used for analysis of prognostic value of deficient DNA mismatch repair (dMMR)
**Table S2.** Tumour‐infiltrating lymphocyte density by marker and compartment according to cancer type and MMR status

## Data Availability

TCGA data used in this study are available for download from the NCI Genomic Data Commons Data Portal: https://portal.gdc.cancer.gov. Other data used in this publication will be shared subject to ethical permissions upon reasonable request addressed to the corresponding author: david.church@well.ox.ac.uk.
