# Supplementary material for: Discordant prognosis of mismatch repair deficiency in colorectal and endometrial cancer reflects variation in antitumour immune response and immune escape
Source: J Pathol. 2022 Apr 1;257(3):340–51. doi: 10.1002/path.5894 (PMC9322587; doi:10.1002/path.5894)
Supplement: Supplementary file 1 — Supplementary materials and methods Figure S1. PRISMA diagram showing search strategy used for identification of studies for inclusion in meta‐analysis Figure S2. Deficient DNA mismatch repair predicts improved overall survival in colorectal but not endometrial cancer Figure S3. Intrastromal CD3+ and CD8+, and intraepithelial and intrastromal FoxP3+ cell infiltrate by tumour type and MMR status Figure S4. Immune checkpoint expression by cancer type and MSI status Figure S5. MLH1 promoter methylation and expression by cancer type and MSI status Table S1. Details of studies used for analysis of prognostic value of deficient DNA mismatch repair (dMMR) Table S2. Tumour‐infiltrating lymphocyte density by marker and compartment according to cancer type and MMR status [file PATH-257-340-s001.docx]

**Discordant prognosis of mismatch repair deficiency in colorectal and endometrial cancer reflects variation in antitumour immune response and immune escape**

MA Glaire *et al. J Pathol* DOI: 10.1002/path.5894

Supplementary materials and methods

**Supplementary Figures S1–S5**

**Supplementary Tables S1,S2**

Reference numbers refer to the main text list

# **Supplementary materials and methods**

**Cell lines**

Human endometrial adenocarcinoma cell lines AN3CA, HEC-1A, HEC-108, EFE-184, EN, KLE and SNG-II cells were a kind gift from Konstantin Dedes (University of Zurich, Switzerland). EN, HEC-1B, Ishikawa, MFE-296 and NOU1 cells were shared by Britta Weigelt (previously Cancer Research UK, Lincoln’s Inn Fields UK). MFE-280 cells were purchased from the European Collection of Authenticated Cell Cultures (Porton Down, UK). HEC-251 and HEC-59 cells were obtained from the JCRB cell bank (Osaka, Japan). All cell lines were culture in an atmosphere of 5% CO_2_ at 37 °C. SNG-II and AN3-CA were cultured in DMEM medium with 10% foetal bovine serum (FBS); EN in DMEM with 20% FBS; MFE-280 and MFE-296 in 40% RPMI/40% DMEM with 20% FBS; KLE and Ishikawa in DMEM/F12 with 10% FBS; HEC-1-A and HEC-1-B in MEM with 10% FBS; NOU1, HEC-59 HEC-251 and HEC-108 in MEM with 15% FBS, and EFE-184 in RPMI with 10% FBS. MSI status was determined by Promega MSI analysis system and *JAK1* mutation usig WES done in-house or by the Cancer Cell Line Encylopedia: [**https://depmap.org/portal/ccle/**](https://depmap.org/portal/ccle/)

**Interferon-gamma stimulation**

Cells for interferon-gamma (IFN-$\boldsymbol{\gamma}$) stimulation were plated at 1x10^6^ cells in 25 cm^2^ cell culture flasks, grown to 70–80% confluency, and serum starved for 24 h before treatment with IFN-$\boldsymbol{\gamma}$ (554617, BD Biosciences, Franklin Lakes, NJ, USA) at 75 ng/ml or anequivalent volume of PBS for 16 h before cell lysis and protein collection.

**Western blotting**

Cells for immunoblotting were washed with ice cold PBS and then lysed in RIPA buffer with protease and phosphatase inhibitors (Pierce Halt Cocktail, ThermoFisher, Waltham, MA, USA). Lysate protein concentration was determined using the CB-XTM Protein Assay (GBiosciences, Saint Louis, MO, USA). Proteins (30 μg) from each sample were separated using NuPAGE 3-to-8%, Tris-Acetate gels (Invitrogen, Waltham, MA, USA) and blotted onto PVDF membranes (Millipore, Burlington, MA, USA) by wet transfer. Membranes were blocked with 5% dried milk in 0.1% TBST for 1 h, incubated with primary antibody overnight at 4 °C followed by 1 h at room temperature, washed with TBST x 3, incubated with loading control antibody to GAPDH for 1 h at room temperature, washed x 3 and incubated with anti-mouse IgG/anti-rabbit IgG as appropriate (1:15000 in 5% dried milk at room temperature for 1 h. Membranes were then imaged using the LI-COR imaging system (LI-COR Bioscience, Lincoln, NE, USA). Antibodies used were: anti-human JAK1 antibody (AB_397627, BD Biosciences 1:500), anti-human STAT1 antibody (AHO0832, Thermo Fisher Scientific, 1:500), anti-human Phospho-STAT1 antibody (33-3400, Thermo Fisher Scientific, 1:500), anti-human HLA Class I Heavy Chain antibody (MUB2037P, Nordic MUbio (Susteren, NL), 1:1000), anti-human GAPDH antibody (5174, Cell Signaling Technology (Beverly, MA, USA), 1:1000). Secondary antibodies: LI-COR IRDye 680RD Goat Anti-Mouse IgG (LIC-926-68070, LI-COR Bioscience, 1:15000) and LI-COR IRDye 680RD Goat Anti-Rabbit IgG (LIC-925-68071, LI-COR Bioscience, 1:15000).

**The packages used for analyses were:**

Salmon: Patro *et al*, 2017 [51] <https://combine-lab.github.io/salmon/>

TCGA Biolinks: Colaprico *et al*, 2016 [52] <https://bioconductor.org/packages/release/bioc/html/TCGAbiolinks.html>

DESeq2: Love *et al*, 2014 [53] <https://bioconductor.org/packages/release/bioc/html/DESeq2.html>

ClusterProfiler: Yu *et al*, 2012 [54] <https://bioconductor.org/packages/release/bioc/html/clusterProfiler.html>

Meta: Schwarzer, 2015 [55] <https://cran.r-project.org/web/packages/meta/index.html>

Ggplot2:Wickham, 2016 [56] <https://cran.r-project.org/web/packages/ggplot2/index.html>

MAFtools: Mayakonda *et al*, 2018 [57] <https://bioconductor.org/packages/release/bioc/html/maftools.html>

# **Figure S1. PRISMA diagram showing search strategy used for identification of studies for inclusion in meta-analysis**

MMR – DNA mismatch repair; MSI – microsatellite instability; DFS – disease-free survival’ CRC – colorectal cancer; EC – endometrial cancer; OS – overall survival

# **Figure S2. Deficient DNA mismatch repair predicts improved overall survival in colorectal but not endometrial cancer**

Forest plot showing meta-analysis of clinical trials which have examined association of dMMR with overall survival (OS) in CRC and EC (details in methods). *pooled analysis includes Mayo Clinic and North Central Cancer Treatment Group (NCCTG) trials 78-48-52, 84-46,52/Intergroup 0035, 89-46-51, 79-46-04, 87-46-51, 91-46-53, Federation Francophone de la Cancerologie Digestive (FFCD) 8802, Gruppo Italiano Valutazione Interventi in Oncologia (GIVIO), National Cancer Institute of Canada (NCIC) C03, NSABP C-01, C-02, C-03 and C-04. Superscript numbers show main text reference list numbers.

**
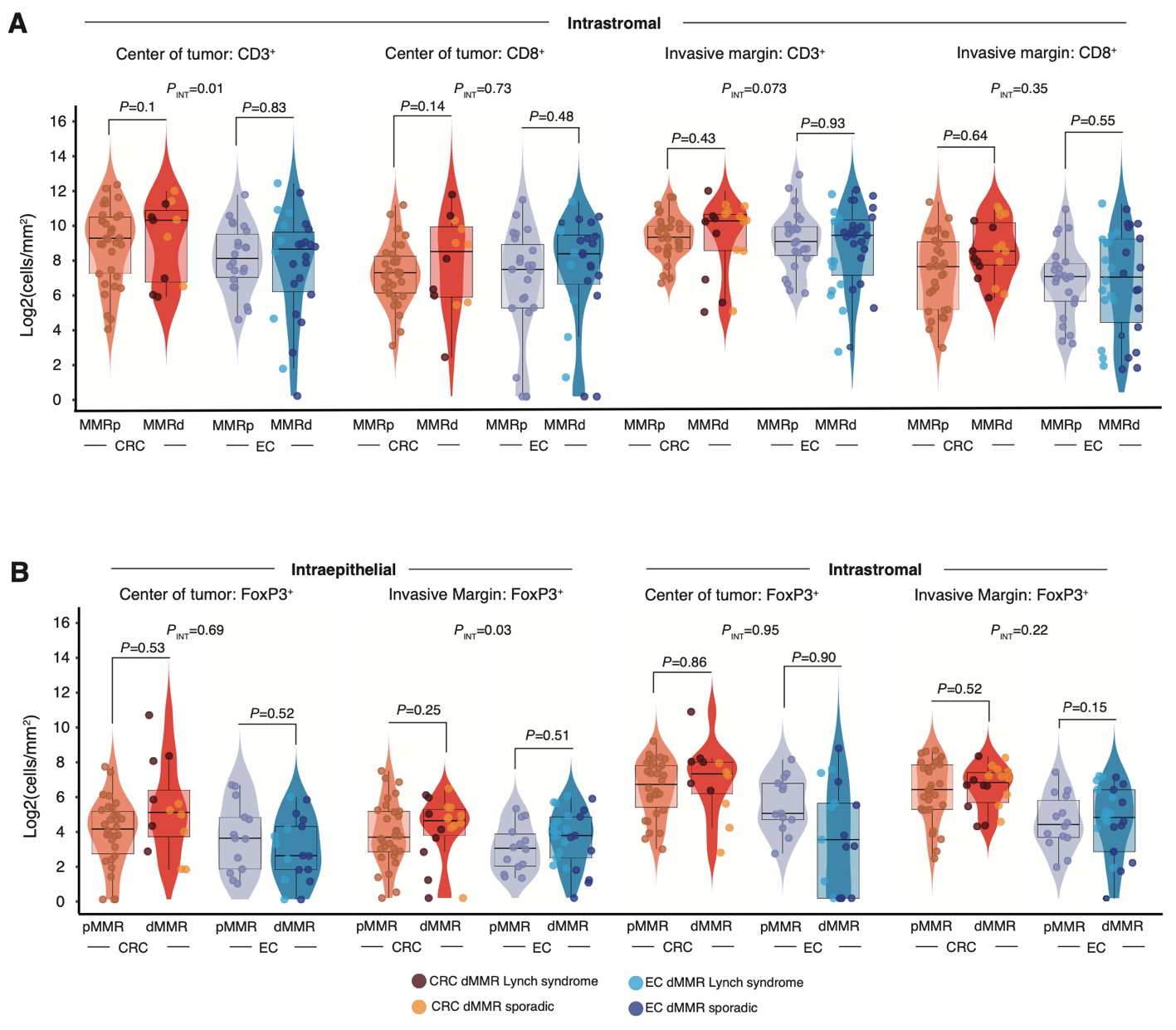
**

# **Figure S3. Intrastromal CD3^+^ and CD8^+^, and intraepithelial and and intrastromal FoxP3^+^ cell infiltrate by tumour type and MMR status**

(A) Quantification of density of intrastromal CD3^+^ and CD8^+^ cells in the tumour centre and invasive margin. (B) Quantification of density of intraepithelial (left panels) and intrastromal (right panels FoxP3^+^ cells in the tumour centre and invasive margin. Comparison between groups used Mann–Whitney U tests (tests for interaction by aligned rank ANOVA are shown in supplementary material, Table S1).

# **Figure S4. Immune checkpoint expression by cancer type and MSI status**

Gene level estimates of immune checkpoint expression in transcripts per million (TPM). Comparison between groups used Mann-Whitney U tests. Tests for interaction used aligned rank ANOVA. MSS – microsatellite stable; MSI – microsatellite unstable.

# **Figure S5. *MLH1* promoter methylation and expression by cancer type and MSI status**

(A) *MLH1* promoter methylation by cancer type and MSI status (results shown for the most differentially methylated probe). (B) Corresponding results for MLH1 expression. MSS – microsatellite stable; MSI – microsatellite unstable.

# **Table S1. Details of studies used for analysis of prognostic value of deficient DNA mismatch repair (dMMR)**

|  | **Disease stage (AJCC)** | **Comparative interventions** | **MMR/MSI testing** | **Year of publication** | **Reference** |
| --- | --- | --- | --- | --- | --- |
| **Colorectal** |  |  |  |  |  |
| QUASAR | II-III | FU versus obs | IHC | 2011 | [26] |
| QUASAR2 | II-III | Cap versus cap + bev | IHC | 2016 | [20] |
| VICTOR | I-III | Rofecoxib versus placebo | MSI | 2013 | [27] |
| NCCTG pooled | II-III | Various | IHC & MSI | 2011 | [28] |
| CALGB  89803  9581 | III  II | FU versus IFL  Edrocolomab versus obs | IHC & MSI | 2011 | [29] |
| IDEA | III | 6 versus 3 mo FOLFOX | IHC | 2020 | [30] |
| PETACC 03 | II-III | FU versus FU + iri | MSI | 2009 | [31] |
| PETACC 08 | III | FOLFOX versus FOLFOX + cetuximab | IHC | 2016 | [32] |
| ASI | II-III | Tumour cell vaccine versus obs | MSI | 2012 | [33] |
| CKVO 90-11 | III | FU-L versus FU-LL | MSI | 2005 | [34] |
| NSAS-CC & NSAS-RC | III | UFT versus obs | IHC | 2016 | [35] |
| MOSAIC | II-III | FU versus FOLFOX | IHC | 2015 | [36] |
| **Endometrial** |  |  |  |  |  |
| PORTEC  -1  -2 | I  I/IIA | EBRT versus obs  VBT versus EBRT | IHC | 2016 | [37] |
| PORTEC-3 | I-III | CTRT versus EBRT | IHC | 2020 | [38] |
| GOG-210 | I-IV | NA  (observational study of molecular & pathological staging | IHC & MSI | 2017 | [39] |
| NCIC-EN5 | I-II | RT versus obs | MSI | 2010 | [40] |

AJCC – American Joint Committee on Cancer; MMR – DNA mismatch repair; MSI – microsatellite instability; FU – fluorouracil; obs – observation; IHC – immunohistochemistry; Cap – capecitabine; bev – bevacizumab; IFL – irinotecan fluorouracil leucovorin; FOLFOX – fluorouracil, leucovorin, oxaliplatin; FU-L – fluorouracil, leucovorin; FU-LL – fluorouracil, leucovorin, levamisole; UFT – tegafur-uracil; EBRT – external beam radiotherapy; VBT – vaginal vault brachytherapy; CTRT – combined adjuvant chemotherapy and external beam radiotherapy; RT – radiotherapy.

# **Table S2. Tumour-infiltrating lymphocyte density by marker and compartment according to cancer type and MMR status**

|  | **Colorectal Cancer** | | | | | | **Endometrial cancer** | | | | | |  |
| --- | --- | --- | --- | --- | --- | --- | --- | --- | --- | --- | --- | --- | --- |
| **Marker-compartment** | pMMR | dMMR sporadic | dMMR LS | *P*  (pMMR versus dMMR SP) | *P*  (pMMR versus dMMR LS) | *P*  (pMMR versus dMMR all) | pMMR | dMMR sporadic | dMMR LS | *P*  (pMMR versus dMMR SP) | *P*  (pMMR versus dMMR LS) | *P*  (pMMR versus dMMR all) | *P*_INTERACTION_  (cancer type*MMR status) |
| **Centre tumour** |  |  |  |  |  |  |  |  |  |  |  |  |  |
| CD3+ intraepithelial | 110.5  (20.2–246.1) | 738.6  (189.9–1099.1) | 837.0  (270.1–1380.5) | 3.37e-05 | 0.017 | 1.2e-04 | 74.1  (44.1–177.8) | 168.5  (61.2-245.2) | 144.9  (52.8–380.0) | 0.50 | 0.32 | 0.39 | 4.3e-04 |
| CD8+ intraepithelial | 61.8  (12.4–190.6) | 218.1  (125.1 -586.8) | 243.9  (113.1–665.2) | 0.015 | 0.029 | 8.8e-03 | 55.6  (29.1–115.6) | 61.5  (15.5–158.9) | 135.1  (59.4–348.6) | 0.98 | 0.059 | 0.41 | 7.3e-03 |
| FOXP3+ intraepithelial | 20.3  (10.2–73.9) | 41.0  (34.7–54.8) | 18.4  (9.9–88.9) | 0.27 | 0.97 | 0.53 | 13.7  (3.4–31.2) | 15.5  (2.2–44.2) | 21.5  (13.0–43.9) | 0.89 | 0.12 | 0.52 | 0.69 |
| CD3+ intrastromal | 571.4  (309.3–782.3) | 926.7  (418.4–1229.1) | 826.2  (127.1–1013.0) | 1.8e-03 | 0.80 | 0.10 | 488.8  (213.5–782.7) | 508.7  (330.9–1051.2) | 566.0  (214.1–1141.4) | 0.61 | 0.87 | 0.83 | 0.01 |
| CD8+ intrastromal | 176.6  (34.0–542.7) | 341.7  (208.0–1473.5) | 287.4  (252.7–529.3) | 0.08 | 0.20 | 0.14 | 112.4  (49.5–230.8) | 116.0  (21.7–244.2) | 206.6  (95.8–801.2) | 0.83 | 0.15 | 0.48 | 0.73 |
| FOXP3+ intrastromal | 181.4  (77.0–487.8) | 283.5  (139.6–393.4) | 169.2  (116.1–288.4) | 0.75 | 0.96 | 0.86 | 46.8  (30.3–102.7) | 42.5  (16.9–170.5) | 87.6  (21.7–244.2) | 0.63 | 0.68 | 0.90 | 0.95 |
| **Invasive margin** |  |  |  |  |  |  |  |  |  |  |  |  |  |
| CD3+ intraepithelial | 95.6  (19.3–373.9) | 1114.3  (366.0-3185.6) | 448.7  (221.0–1205.3) | 1.0e-03 | 0.12 | 3.3e-03 | 37.3  (15.2–146.2) | 44.9  (21.3–168.5) | 93.4  (14.8–240.9) | 0.94 | 0.48 | 0.78 | 3.1e-04 |
| CD8+ intraepithelial | 56.3  (10.0–172.3) | 515.0  (297.6–556.0) | 150.3  (32.0–257.0) | 4.6e-03 | 0.70 | 0.02 | 36.4  (17.9–122.5) | 91.9  (25.1–154.7) | 89.  (48.3–217.5) | 0.16 | 0.26 | 0.20 | 0.037 |
| FOXP3+ intraepithelial | 22.8  (6.9–53.1) | 32.2  (7.2–51.7) | 295.0  (41.7–599.0) | 1.0 | 0.036 | 0.25 | 14.7  (2.9–39.2) | 6.1  (2.8–41.4) | 11.1  (3.33–19.5) | 0.56 | 0.49 | 0.51 | 0.03 |
| CD3+ intrastromal | 546.0  (131.3–129.5) | 1143.1  (573.7–2397.7) | 614.2  (69.1 – 1231.1) | 0.018 | 0.69 | 0.43 | 246.4  (113.9-656.4) | 160.3  (87.4-431.2) | 497.2  (21.0–1488.8) | 0.86 | 0.62 | 0.93 | 0.073 |
| CD8+ intrastromal | 140.3  (62.1–271.0) | 449.5  (140.7–721.9) | 358.4  (59.6–1092.0) | 0.32 | 0.89 | 0.64 | 262.1  (33.5–446.6) | 300.2  (119.1–563.9) | 254.8  (36.6–816.9) | 0.31 | 0.48 | 0.55 | 0.35 |
| FOXP3+ intrastromal | 174.4  (62.0–407.6) | 164.8  (35.2–288.1) | 476.6  (253.0 – 541.8) | 0.47 | 0.078 | 0.52 | 45.5  (33.9–180.2) | 9.4  (0.0–63.9) | 60.8  (7.2–180.4) | 0.06 | 0.64 | 0.15 | 0.22 |

Values represent sample medians with IQR in parentheses. *P* values were calculated using non-parametric Mann-Whitney U tests. Tests for interaction were performed using aligned rank ANOVA. dMMR sporadic (dMMR SP)– MLH1 methylated; dMMR LS – Lynch Syndrome; dMMR all – combined sporadic and Lynch Syndrome
